# Noise Effects on Health in the Context of Air Pollution Exposure

**DOI:** 10.3390/ijerph121012735

**Published:** 2015-10-14

**Authors:** Stephen A. Stansfeld

**Affiliations:** Centre for Psychiatry, Wolfson Institute of Preventive Medicine, Barts and the London School of Medicine and Dentistry, Queen Mary University of London, Charterhouse Square, London EC1M 6BQ, UK; E-Mail: s.a.stansfeld@qmul.ac.uk

**Keywords:** environment, noise, transport, air pollution, burden of disease, aircraft, road traffic, cohort studies, DALYs

## Abstract

For public health policy and planning it is important to understand the relative contribution of environmental noise on health compared to other environmental stressors. Air pollution is the primary environmental stressor in relation to cardiovascular morbidity and mortality. This paper reports a narrative review of studies in which the associations of both environmental noise and air pollution with health have been examined. Studies of hypertension, myocardial infarction, stroke, mortality and cognitive outcomes were included. Results suggest independent effects of environmental noise from road traffic, aircraft and, with fewer studies, railway noise on cardiovascular outcomes after adjustment for air pollution. Comparative burden of disease studies demonstrate that air pollution is the primary environmental cause of disability adjusted life years lost (DALYs). Environmental noise is ranked second in terms of DALYs in Europe and the DALYs attributed to noise were more than those attributed to lead, ozone and dioxins. In conclusion, in planning and health impact assessment environmental noise should be considered an independent contributor to health risk which has a separate and substantial role in ill-health separate to that of air pollution.

## 1. Introduction

A wide range of environmental stressors have an impact on the health of children and adults. Understanding which pollutants have the greatest magnitude of effect on health can have implications for designing suitable preventive and therapeutic interventions. In the last ten years a number of large scale studies of environmental noise and health have been carried out [1]. In parallel, studies have been published examining the associations of air pollution with health [2]. Because transport sources, such as road traffic, are responsible for both noise exposure and air pollution there has also been an interest in understanding the relative contribution of noise exposure and air pollution to health. Recent studies have strengthened the evidence base for noise and health, beyond effects on noise annoyance and sleep, to providing evidence of convincing health impacts in terms of hypertension, risk of ischaemic heart disease and mortality [3,4,5,6,7,8,9,10,11,12,13,14,15,16,17,18,19,20,21]. In terms of public health, and for practical use in health impact assessment, it would be helpful to understand the relative contribution of these different environmental stressors to health outcomes. In this context, this paper examines the evidence for the contribution of environmental noise exposure, largely road, rail and aircraft noise on health, relative to air pollution.

## 2. Method

A narrative review was carried out without specifying a time limit for the study search. This involved an initial PubMed search on “noise, air pollution and health”. “Health” was not further defined in this initial search but the studies found largely relate to cardiovascular disease. Studies of respiratory disease were not found as noise, unlike air pollution, has not been related to this health outcome. This was supplemented by access to recent reviews of noise and health and additional papers revealed by citation tracking. Initially, additional searches were carried out for “environmental noise and pesticides”, “environmental noise and heavy metals”, “environmental noise and endocrine disrupting chemicals”, “environmental noise and climate change”. There were very few relevant papers on noise and pesticides, heavy metals, endocrine disrupting chemicals or climate change. In these papers there was insufficient evidence to judge the relative contribution of noise and these environmental stressors on health. Thus the focus of the paper has been confined to studies including environmental noise and air pollution exposure. Environmental noise is defined in this paper as noise from aircraft, road traffic and railways—the main sources of outdoor noise assessed in these studies. Occupational studies and studies of hearing loss have been excluded. Twenty five primary research studies were identified that included assessments of both air pollution and environmental noise and health outcomes. The characteristics and results of studies of noise and air pollution exposure and hypertension, atherosclerosis, ischaemic heart disease, stroke and mortality have been reported in a table (Table 1) to aid comparability. Table 2 reports studies of environmental noise, air pollution and children’s cognition and blood pressure. Mental health and cognitive outcomes in adults are also briefly touched on. Quality of the primary studies was assessed in terms of population representativeness, objective measurement of noise and air pollution exposures, sufficient adjustment for confounding factors and objective measurement of health outcomes.

**Table 1:** Studies of air pollution and noise: effects on health.

Reference	Noise Exposure	Air Pollution Exposure	Health Outcome	Sample	Adjustments	Direction of Evidence
**Hypertension and Atherosclerosis**						
De Kluizenaar *et al.* 2007 [6]	RTN at most exposed façade, L_den_ using SKM2 model Exposure and transmission path assessed	Regional background data on PM_10_ and modelling of local road traffic to give annual averages	Medication for hypertension in Groningen sample. Measured hypertension BP >140/90 in PREVEND sample	Cross sectional survey of longitudinal cohort study 40,856, 28–75 years Groningen-self report BP medication 8952 screening clinic visit—measured hypertension	Age, sex, SES, fh of CVD, smoking. Additionally for PREVEND: BMI, plasma cholesterol, level of education	For self-reported hypertension OR = 1.31 95% CI 1.25, 1.37 per 10dB(A) increase in L_den_; In fully adjusted model OR = 1.03 95% CI 0.96, 1.11. In 45–55 year age group fully adjusted OR = 1.19 95% CI 1.02, 1.40 including PM_10_. For those exposed >55 dB(A) OR = 1.31 95% CI 1.08,1.59 adjusting for PM_10_. In PREVEND in 45–55 year age group measured hypertension OR = 1.39 95% CI 1.08, 1.77. No differences in men and women
Fuks *et al*. 2011 [7]	RTN Weighted L_den_	PM_10_, PM_2.5_	Blood pressure (SBP,DBP). Measured hypertension BP >140/90	Cross sectional survey. 4291, 45–75 years Heinz Nixdorf Recall Study	Smoking, alcohol use, physical activity, diabetes mellitus, social and employment status, daily changes in PM, O3 and temperature	Interquartile increase in PM_2.5,_ increase in SBP 1.4 95% CI 0.5–2.3, DBP 0.9 95% CI 0.4–1.4 adjusting for RTN. RTN>65dB(A) Hypertension OR = 1.28, 95% CI 1.04–1.59
Sørensen *et al*, 2011 [8]	RTN SOUNDPLAN using Nordic prediction model from 5 years prior to enrolment to 2000–2002. LAeq at most exposed façade, expressed as L_den_. Railway noise LA _eq24_ 1993–2000	NOx modelled at each address using AirGIS from 5 years prior to enrolment to 2000–2002	Questionnaire reported hypertension. Measured SBP, DBP	Cross sectional and prospective analyses from cohort study 44,083 out of 160,725, 50–64 years from Diet, cancer and health cohort, Copenhagen, Aarhus	Age, sex, calendar year, area of residence, length of education, SES, BMI, smoking, alcohol intake, leisure time sport, air pollution measured as time weighted average of NOx exposure, mean ambient temperature, humidity, season	RTN: 10% highest exposed had a 0.79 mm Hg (95% CI: −0.04; 1.62) and a 0.85 mm Hg (95% CI: 0.02; 1.67) higher systolic BP compared with the lowest exposure group for 1-year and 5-mean 0.26 (95% CI: −0.11; 0.63) mm Hg higher level of SBP per 10 dB(A) higher level of road traffic noise (1-year mean). No associations between road traffic noise and diastolic BP RTN and BP only associated in men and in over 60s. No prospective association between RTN and self-reported hypertension in sample of 32,635. Exposure to railway noise associated with 8% (95% CI: −2%; 19%, *p* = 0.11) higher risk of hypertension
Dratva *et al.* 2011 [5]	Rail RTN Day Night dB(A) for 10 × 10 m grids. Rail noise measured within 1000m	Av annual PM_10_ at residence predicted by dispersion modelling NO_2_ using a hybrid model	Measured SBP, DBP Measured hypertension BP >140/90	Cross sectional analyses in a cohort study 6450 SAPALDIA 2. 28–72 years 2002/2003 Switzerland	Physician diagnosed illness: hypertension, MI, stroke, diabetes, kidney disease, hearing deficit, antihypertensive drugs, smoking, physical activity, BMI, age, education, employment status, work-related exposures, housing characteristics, age of building, years of residency	Significant effect estimates for a 10 dB(A) increase in railway noise during the night SBP β = 0.84;95% CI 0.22, 1.46; DBP β = 0.44; 95% CI 0.06, 0.81 and day (SBP β = 0.60; 95% CI: 0.07, 1.13). Adjustment for NO_2_ left effect estimates almost unchanged. Stronger associations in participants with chronic disease. Significant associations with traffic noise only in participants with diabetes: β = 3.7 95% CI (–0.09, 7.57) *p* = 0.056
Foraster *et al.* 2014 [9]	RTN L_night_ inside at geocoded address adjusted for questionnaire measured insulation	NO_2_ with land use regression model	Hypertension BP > 140/90 Use of antihypertensives	Cross sectional analysis of cohort baseline data 2067 36–82 years REGICOR, Girona, Spain	Age, age squared, sex, education level, physical activity, diet, alcohol consumption, diabetes, deprivation, railway noise, daily temperature	Indoor L_night_ was associated both with hypertension (OR = 1.06; 95% CI:0.99, 1.13) and SBP (β = 0.72; 95% CI: 0.29, 1.15) per 5 dB(A); and NO_2_ was associated with hypertension (OR = 1.16; 95% CI: 0.99, 1.36), SBP (β = 1.23; 95% CI: 0.21, 2.25), and DBP (β = 0.56; 95% CI: −0.03, 1.14) per 10 μg/m^3^. L_night_ was associated only with hypertension and NO_2_ with BP only
Foraster *et al*. 2014 [10]	RTN Noise model 2005 CadnaA software L_night_	Annual average NO_2_ with land use regression model controlling for short term air pollution with NO_2_ from urban background station	Hypertension BP > 140/90 Use of antihypertensives	Cross sectional survey 3700, 35–83 years Girona	Age, age squared, sex, living alone, education level, BMI, alcohol consumption, diabetes, deprivation, road traffic noise, railway noise, night time noise, daily temperature	Correlation of annual mean NO_2_ with L_night_ *r* = 0.74 10 microgm/m^3^ increase in av annual NO_2_ associated with 1.34 mmHg 95% CI 0.14, 2.55 increase after full adjustment in non-medicated sample. Transportation noise main covariate. SBP per 10-dB(A) change in L_night_ in the model for nonmedicated participants were β = −0.94 (95% CI: −2.53, 0.64, *p* = 0.244) (traffic noise) and β = −0.21 (95% CI: −0.63, 0.21, *p* = 0.326) (railway noise) Stronger associations of air pollution and BP in those with existing CVD. Interaction between NO_2_ and SBP and noise such that individuals exposed to traffic L_night_ ≥ 55 dB(A) (β = 1.82; 95% CI: 0.56, 3.07) compared with those exposed to lower noise levels (β = −0.39; 95% CI: −2.17, 1.39), *p* for interaction = 0.03.
Babisch *et al*. 2014 [11]	RTN L_dn_ at most exposed façade, noise maps using CADNA/A software. Also rail noise	Modelled annual average PM_2.5_ using land use regression models	Measured BP, isolated systolic hypertension. Hypertension. BP > 140/90. Antihypertensive medication	Cross sectional 4166, 25–74 years KORA Study Oct 1999–April 2000. RR = 67% 2 samples: City of Augsburg 1893; Greater Augsburg 2273	Age, sex, smoking, alcohol consumption, BMI, physical activity, individual and area level SES	Traffic noise Hypertension OR = 1.11 95% CI 0.94, 1.30 adjusting for PM_2.5._ In 894 longer term residents OR = 1.12 95% CI 0.90, 1.49 adjusting for PM_2.5_. City of Augsburg, *n* = 1601, isolated hypertension OR = 1.43 95% CI 1.10, 1.86 adjusting for PM_2.5_. 1 microgram/m^3^ increase in PM_2.5_ OR = 1.11, 95% CI 0.98, 1.27 after adjustment for noise_._ Traffic noise and air pollution no longer significant after mutual adjustment
Kälsch *et al*. 2014 [12]	RTN façade levels, 24 h mean L_den,_ L_night_	EURAD-CTM model for PM_2.5_. PM_10_ on a scale of 1 km^2^	Thoracic aortic calcification using cardiac electron beam CT scanning	Cross sectional 4238. Mean age 59.6 ± 7.8 years. Heinz Nixdorf Recall Study. Baseline data 2000–2003	Education, income, neighbourhood unemployment, smoking, environmental tobacco smoke, physical activity, alcohol intake, anthropometry, BP, diabetes, current medication	PM_2.5_ associated with increased thoracic aortic calcification of 18.1% 95% CI 6.6, 30.9%. L_night_ associated with increased thoracic aortic calcification of 3.9% 95% CI 0.0, 8.0%. Both analyses mutually adjusted. No effect modification
**Cardiovascular Morbidity**						
Selander *et al*. 2009 [13]	Residential exposure 1970–1992, 1994. Nordic Prediction model. RTN, ACN and occupational noise	Dispersion methods, historical data on RT emissions	First non-fatal, fatal myocardial infarction (MI)	Population based case control study 1571 with MI, 2095 controls 45–70 years	Sex, age, catchment area, diabetes, physical activity, air pollution, occupational noise exposure	For RTN, MI OR = 1.12 95% CI 0.95, 1.53; Excluding other noise sources and hearing loss OR = 1.38 95% CI 1.11, 1.71. No effect modification by sex or air pollution. Adjustment for air pollution reduced the coefficient by 7%. Air pollution and RTN correlated *r* = 0.6
Sørensen *et al.*, 2012 [14]	RTN SoundPLAN 1990, 1995, 2000, 2005, Nordic Prediction Model L_den_	NO_x_ Air GIS model Urban background calculated by area source dispersion model	First incident MI 1600. Included sudden cardiac death	Prospective cohort study 57,053. 50,614 in analytic sample. 50–64 years enrolled in 1993-1997. Mean FU 9.8 years	Age, sex, education, smoking, fruit and vegetable intake, alcohol, physical activity, BMI, calendar year railway, airport noise. In further model measured BP cholesterol and diabetes	RTN L_den_ IRR = 1.12 per 10 dB(A) year exposure at diagnosis 95% CI 1.02, 1.22 adjusting for NO_x_. 5 year time weighted mean exposure prior to event IRR = 1.12 95% CI 1.02, 1.23. Still 10% increased risk in further model adjusting for BP, cholesterol and diabetes. NO_x_ showed similar trends but was not significantly associated with MI
Hart *et al*. 2013 [15]	RTN Distance to major roads <50 m defined as close.	To examine changes in distance to road, each consecutive pair of addresses was categorized: (1) consistently close; (2) consistently far; (3) change from close to far; and (4) change from far to close. Difference in ambient NO_2_ between each pair of addresses	Incident non-fatal and fatal MI (2948) All-cause mortality (11,502)	Longitudinal cohort study. Nurses Health Study 84,562 out of 121,700 female nurses 30–55 years in 1976	Age, race, individual SES, physical activity, BMI, alcohol use, diet, smoking, Hypertension, physician diagnosed diabetes, fh of MI	Proximity to roads: MI HR = 1.11 95% CI 1.01, 1.22. All cause mortality HR = 1.05 95% CI 1.00, 1.10. Moving closer to traffic MI HR = 1.50 95% CI 1.11, 2.05. All cause mortality HR = 1.17 95% CI 1.00, 1.37. One ppb increase in NO_2_ MI HR = 1.22 95% CI 0.99, 1.50. All cause mortality HR = 1.03 95% CI 0.92, 1.15.
Floud *et al.* 2013 [16]	ACN, RTN Modelled aircraft noise contours Common noise models with 1 dB(A) resolution Road Traffic noise maps 2002 reference year. Expressed as _,_ L_night,_ L_Aeq16hr_	Ambient NO_2_ in UK, Netherlands and Sweden Assessed around 3 airports NO_2_ using APMoSPHERE models *n* = 4000. Mean annual values at place of residence	Self-reported Dr-diagnosed Angina pectoris, MI and stroke 276 events	Cross sectional survey 4712 HYENA Study 45–70 years	Age, sex, BMI, alcohol intake, physical activity, education, smoking, ethnicity	Night time ACN, heart disease and stroke OR = 1.25 95% CI 1.03, 1.51 per 10 dB (A). For those resident ≥ 20 years adjusting for exposure to air pollution. 24 h average RTN, heart disease and stroke OR = 1.19 95% CI 1.00, 1.41 but adjustment for air pollution suggested this may have been due to confounding by air pollution.
De Kluizenaar *et al*. 2013 [17]	RTN calculated using SKM2. Emission and transmission calculated. Expressed as L_den_	Air pollution at most exposed façade. Dutch National Air Quality Monitoring Network. 1 km × 1 km annual average PM_10_ NO_2_	Hospital admissions for IHD, Cerebrovascular disease	Prospective cohort study 18,973 residents of Eindhoven GLOBE Study 15–74 years	Age, gender, marital status, education, smoking, alcohol use, physical activity, BMI, employment status, financial problems, history of CVD	For 10 dB(A) increase in L_den_ RR = 1.12 95% CI 1.04, 1.21 after adjustment non-significant RR = 1.01 95% CI 0.94, 1.09 and additionally PM_10_ RR = 1.00 95% CI 0.91, 1.10 Similar findings for cerebrovascular disease. For PM_10_ RR = 1.06 95% CI 1.01, 1.11 after full adjustment including L_den_ RR = 1.01 95% CI 0.95, 1.08. Similar findings for elemental carbon and NO_2_
Correia *et al*. 2013 [18]	ACN contours from US FAA. Integrated noise model version 7A	PM_2.5_ Ozone. For 1165 and 779 zip codes out of 2218 zip codes. EPA Air Quality database.	ICD-9 coded CVD admissions	Ecological small area study. 6,027,363 of US population, >65 years, eligible for Medicare residing near 89 regional airports in 2009	Age, sex, race, zip code level SES, roadway density	For 90^th^ centile of noise exposure a 10 dB(A) increase resulted in 2.9, 95% CI 0.8%, 5.0%, including air pollution increase of 3.5, 95% CI 0.2, 7.0% in relative rate of CVD hospitalization. In zip codes with air pollution data, 6.8% of CVD hospitalizations attributable to fine particulate matter and 4.2% to ozone. Population attributable fraction for noise in the subset of zip codes with air pollution data was 2.2%
Sorensen *et al*. 2014 [19]	RTN Soundplan Nordic Prediction Model L_Aeq_ expressed as L_den_	Ambient NO_x_, NO_2_ at residence. AirGIS 1987–2009	Non-fatal and fatal incident Stroke cases validated by physician review 1999 cases	Prospective cohort study. 57,053 enrolled in 1993–1997, 50–64 year Copenhagen/Aarhus. Mean FU 11.2 years	Sex, length of school attendance, area SES, smoking, fruit and vegetable intake, alcohol, coffee, physical activity, BMI, calendar year	Higher mean annual exposure at time of diagnosis of 10 mg/m^3^ NO_2_ and 10dB(A) RTN was associated with ischemic stroke IRR = 1.11 95% CI 1.03, 1.20% and1.16 95% CI 1.07,1.24 in single exposure models. In two-exposure models RTN IRR = 1.15, 95% CI 1.04, 1.26 and not NO_2_ IRR = 1.02 95%CI 0.92, 1.12 was associated with ischemic stroke. Strongest association for combination of high noise and high NO_2_ IRR = 1.28 95% CI 1.09, 1.52. Fatal stroke associated with air pollution not traffic noise.
**Mortality**						
Beelen *et al*. 2009 [2]	RTN EMPARA noise model to 25 × 25 resolution 2000–2001 data	Black smoke, NO_2_, PM_2.5_ Sum of regional, urban and local traffic using regression models	Cardiovascular mortality, including Heart failure, cerebrovascular mortality from 1987–1996	Prospective cohort study 120,852 55–69 years from Netherlands Cohort study on cancer	Age, sex, smoking status, neighbourhood SES, local area (COROP score)	Road Traffic noise and black smoke correlated *r* = 0.24. Black smoke: cerebrovascular RR = 1.39 95% CI 0.99, 1.94; heart failure RR = 1.75 95% CI 1.00, 3.05- not affected by adjustment for RTN. Traffic noise > 65 dB(A) IHD RR = 1.15 95% CI 0.86,1.53; heart failure RR = 1.99 95% CI 1.05, 3.79 reduced by adjustment for black smoke (RR = 1.90 95% CI 0.96–3.78). Similar RRs for NO_2_ and PM_10_. No difference in men and women
Huss *et al*. 2010 [3]	ACN L_dn_ Yearly av exposure to aircraft noise: Zurich airport dedicated noise exposure model, resolution 100 × 100 m. Model from Federal Office of civil aviation for other 64 airports	Background air pollution dispersion models, resolution 200 × 200 m and proximity to major roads	Deaths from acute MI and circulatory disease, 15,532 deaths from MI	Prospective cohort study 4.6 million, Swiss National Cohort followed end of 2000–2005 30 years plus	Sex, education, marital status, Swiss or other, municipality SES, type of building, distance to major roads, PM_10,_ urbanicity.	For ACN > OR = 60dB(A) HR = 1.3 95% CI 0.96,1.7 adjusting for PM_10_. For those resident > 15 years HR = 1.5 95% CI 1.0, 2.2. No associations between ACN and all-cause or stroke mortality. Lung cancer associated with PM_10_ and proximity to major roads
Gan *et al*. 2011 [20]	RTN Cadna A model L_den_ dB(A) at postcode. Annual av noise level 63.4 dB(A)	NO_2_, NO, Black carbon, PM_2.5_ using land use regression models in 2003	CHD mortality from Provincial Death Registry 3095 deaths	Prospective cohort study 445,368 Vancouver residents 45–85 years, 5 year exposure period January 1994–December 1998, 4 year follow up January 1999–December 2002	Age, sex, neighbourhood SES, COPD, hypertensive heart disease	Equal to interquartile ranges, noise 6, 95% CI 1,11. Black carbon 4, 95% CI 1,8. RTN: Highest noise decile 33 95% CI 4, 43 for CHD mortality compared to lowest decile 10 dB(A) elevation in residential noise associated with 9% increase in cardiac mortality. Effect of noise little altered after adjustment for NO_2_ and PM_2.5_ but reduced, still significant after adjustment for black carbon. No exposure response relationship. No interaction between black carbon and noise. Similar effects men and women. No significant effect of aircraft noise (annual average noise level 32dB(A))
Hansell *et al*. 2013 [4]	ACN 10 ×10 m grid ANCON model Weighted annual average noise levels calculated for day and night	PM_10_ at spatial resolution 20m by 20m. Dispersion modelling. London emissions toolkit. London air pollution toolkit.	Hospital admission and mortality for stroke, CHD, CVD 2001–2005. Postcode data on hospital admissions	Small area ecological study 12 London Boroughs around Heathrow airport 3.6 million residents Hospital admissions from 12,110 census output areas Mortality in 2378 super output areas.	Ethnicity, deprivation and lung cancer as smoking proxy	Hospital admissions: statistically significant linear trends of increasing risk with higher levels of both LAeq, 16 h and L_night_) ACN. LAeq, 16 h > 63 dB(A) *v* ≤ 51 dB(A), RR = 1.24 (95% CI 1.08, 1.43 for stroke; RR = 1.21, 95% CI 1.12, 1.31 for CHD; RR = 1.14, 95% CI 1.08, 1.20 for CVD adjusted for age, sex, ethnicity, deprivation, and lung cancer mortality. All robust to adjustment by PM_10_. Stroke mortality RR = 1.21, 95% CI 0.98,1.49 CHD mortality RR = 1.15, 95% CI 1.02, 1.30 for CVD mortality RR = 1.16, 95% CI 1.04, 1.29 Night time ACN RR (>55 dB(A) *vs.* ≤50 dB(A)) = 1.23, 95% CI 1.02, 1.49, 1.11 95% CI 0.99, 1.24 and 1.14 95% CI 1.03, 1.26
Halonen *et al.* 2015 [21]	Annual RTN levels modelled 2003–2010 at geometric centroids of 190,000 postcode locations TRANEX model Expressed as _,_ L_night,_ L_Aeq16hr_	NOx, PM_2.5_ Average 2003–2010 aggregated to LSOA and COA levels using KCL urban dispersion modelling system	CVD admissions All-cause and CVD mortality	Small area ecological study 8.6 million population of London All adults >25 years. Elderly >75 years	Age, sex, area-level deprivation, ethnicity, smoking, neighbourhood spatial structure	Daytime RTN: hospital admission for stroke RR = 1.05 95% CI 1.02, 1.09 in adults. RR = 1.09 95% CI 1.04, 1.14 in elderly in areas >60 *vs.* <55 dB(A) Night time noise associated with stroke admissions only among elderly. Daytime noise: all-cause mortality RR = 1.04 95% CI 1.00–1.07 in adults in areas >60 *vs.* <55 dB(A). Adjustment for air pollution had minimal or no effect on results

RTN = Road traffic noise; ACN = Aircraft noise; BP = Blood Pressure; SBP = Systolic Blood Pressure; DBP = Diastolic Blood Pressure; MI = Myocardial infarction; CHD = Coronary heart disease; CVD = Cardiovascular disease; HR = Hazard ratio; OR = Odds ratio; RR = Relative risk; IRR = Incidence rate ratio SES = Socioeconomic status.

**Table 2:** Environmental noise, air pollution and cognitive outcomes and blood pressure in children.

Reference	Noise Exposure	Air Pollution Exposure	Cognitive/Health Outcome	Sample	Adjustments	Direction of Evidence
Clark *et al*, 2012 [22]	ACN 16 hour outdoor L_Aeq_. 7 am–11 pm, July–September 2000. Outdoor RTN based on proximity to motorways, A & B roads, traffic flow data and confirmed by measurement at school facade	Annual mean ambient NO_2_ Combined emission-dispersion and regression modelling using Kings College London Emissions toolkit	Suffolk Reading Scale Child Memory Scale Search and Memory Task BP	Cross sectional survey using school based sample. 719 children from 22 schools around Heathrow airport. 9–10 years RANCH Study—UK sample	Parental employment status, housing tenure, crowding, maternal education, ethnicity, main language spoken at home. For BP analyses: premature birth, parental high blood pressure, birth weight, cuff size, BMI, ambient temperature	ACN associated with poorer recognition memory (β = −0.045, −0.073, −0.017 <0.01), conceptual memory recall (β = −0.015 95% CI −0.026, −0.003) and reading comprehension (β= −0.012 95% CI −0.023, −0.000063 *p* = 0.05) and information recall (β = −0.043 95% CI −0.086, −0.000036 0.05 adjusting for ambient NO_2_. No effects of NO_2_ on cognition. No effects of noise or NO_2_ on BP
Van Kempen *et al*. 2012 [23]	Modelled ACN 250 × 250 grids expressed in LAeq, 7–23 h from NLR for 2001. RTN from modelled composite data 2000–1 Resolution 25 × 25 grid	Modelled NO_2_ using land use regression models	Neurobehavioral Evaluation System (NES): Reaction time, attention (Switching Attention Test), coordination, Digit Symbol Substitution Test, Digit Memory Span Test	Cross sectional survey using school based sample 553 primary school children 9–11 years RANCH Study-Netherlands	Age, sex, crowding, home ownership, mother’s education, employment, longstanding illness, parental support, main language spoken at home, school window glazing, road and air traffic noise	NO_2_ at school associated with decrease in memory span length measured during DMST (*X*^2^ = 6.8, df1, *p* < 0.01)—remained after additional adjustment for transportation noise. RTN, ACN at school associated with the number of errors made during the “arrow” (*X*^2^ = 7.5, df1, *p* < 0.006) and “switch” (*X*^2^ = 4.8, df1, *p* < 0.028) conditions of the SAT—remained after adjustment for NO_2_. Interaction: children living in high RTN have shorter reaction times as concentration of NO_2_ increases.
Bilenko *et al*. 2015 [24]	RTN EMPARA noise mapping model resolution 25 × 25 m. Expressed as L_den_	Annual mean ambient NO_2_ PM_2.5_ PM_10_ at home and school. Land use regression modelling. Short term air pollution based on previous 7 days from background monitoring sites	SBP, DBP	Cross sectional analyses of a cohort study. 1147 12 years old PIAMA Birth cohort	Age, gender, BMI, cuff size, gestational age at birth, birth weight, physical activity, maternal education, maternal smoking in pregnancy, parental smoking, breast feeding, maternal hypertension, respiratory infections, ambient and room temperature	Interquartile range increase in BP: Long term home and school exposure to NO_2_, PM_2.5_ associated with raised DBP: for NO_2_ adjusted mean difference = 0.83 mm Hg 95% CI 0.06, 1.61 and for PM_2.5_ adjusted mean difference= 0.75%, 95% CI −0.08, 1.58. No effects on SBP or effects of noise

RTN = Road traffic noise; ACN = Aircraft noise; BP = Blood Pressure; SBP = Systolic Blood Pressure; DBP = Diastolic Blood Pressure; DMST = Digit Memory Span Test; DSST = Digit Symbol Substitution Test; SES = Socioeconomic status.

Most studies reviewed here employ noise modelling techniques to assess noise exposure. Noise models produce. “A” weighted energy-equivalent sound levels based on noise sources, the models including the acoustic features of environments through which noise is propagated from the source to a receiver. A calculation method is applied taking into account the environmental features to estimate sound levels at the receiver and produce noise contour maps. Typically outdoor exposures at the most exposed building facades are produced and expressed as L_den_ (weighted averages for day, evening and night with 5 dB penalty for evening and a 10 dB(A) penalty for night time exposure).

Two predominant methods are used for air quality modelling. Dispersion modelling attempts to replicate atmospheric conditions (e.g., wind speed, air temperature) in order to provide estimates of air pollution from an emission source. Land use regression models characterize air pollution exposure for individual locations by employing monitored levels of pollutants as dependent variables in multiple regression analyses in which the independent variables include such variables as traffic and topography. The advantage of this method is that it can account for local site specific variables.

## 3. Results

### 3.1. Correlation between Environmental Noise and Air Pollution Exposure

For road traffic, noise is largely produced by the engine and by the contact of tyres on the ground, while air pollution is emitted from the exhaust from the engine. For aircraft, noise may arise from the engines but also from the aircraft frame and is most prominent for aircraft landing or taking off as well as the noise of the aircraft on the ground. Air pollution from aircraft arises from the plane’s engines. Rail noise arises from the contact of the train wheels with the track, from the locomotive engine, from wind resistance to the train and is often accompanied by vibration. Air pollution arises from train engines, usually diesel fuelled engines, rather than electrically powered trains.

A key consideration in disentangling the associations of noise exposure and air pollution with health is to understand how closely the two exposures are associated. Strong correlations between the two exposures may make it more difficult to separate out the effects of each exposure whereas weak or inconsistent correlations may make geographical separation of the exposures, and thus, the links to health outcomes more feasible. In a Swiss study correlations between road traffic noise and PM_10_ were as low as 0.16 while Pearson’s correlations between night time rail noise and PM_10_ were 0.37 [5]. NO_x_ and L_den_ were moderately correlated in a road traffic noise study (Spearman’s *r* = 0.62) [14]. Despite road traffic vehicles being a source of both noise exposure and air pollution many studies have shown that correlations between noise exposure and air pollution in community studies are often not that high; in a study of metropolitan Vancouver modelled road traffic noise levels were not strongly correlated to land use regression modelled air pollutants [20]. The highest correlations were for black carbon and noise exposure (Spearman’s *r* = 0.44) while the lowest were for PM_2.5_ (Spearman’s *r* = 0.14). Proximity measures to major roads have not been found to be adequate surrogate measures for either sound levels or air pollution [25]. However the strongest correlations were between air pollutants and road traffic noise measured at the roadside and at night rather than during the day [26]. In the Multi-Ethnic Study of Atherosclerosis and Air Pollution (MESA Air) study two week NO and NO_2_ and ultrafine particles were measured in 105 locations near major roads in 9 US communities as well as 5 min “A” weighted sound pressure levels [25]. Sound levels were most closely associated with NO levels but the correlations were not high (*r* = 0.20–0.60). Downwind correlations from major roads were higher (*r* = 0.53–0.74) whereas upwind correlations were lower. Meteorological conditions are more likely to affect air pollution than noise that tends to be more consistent day by day [27]. On the other hand noise exposure will be more affected by intervening buildings and noise barriers and indoor noise exposure will be modified by access to a quiet side of a building for bedrooms and living rooms. Some transportation sources such as aircraft and electrically powered trains may be more likely to cause noise than be a source of air pollution.

There is a common misconception that certain areas of cities, usually indicated by less advantaged socioeconomic position, may be associated with a range of correlated environmental pollutants and psychosocial stressors. This could indicate that in certain less advantaged urban areas the correlations between noise and air pollution may be higher than in more advantaged areas because there is a clustering of noise and air pollution sources e.g. heavily trafficked roads. While this may be the case in some cities it is by no means an invariable finding. For instance, in an area study of New York City using Geographic Information Systems (GIS) technology to examine the association of social stressors, a derived factor of “noise complaints and property crime” was associated with indicators of air pollution (PM_2.5_ NO_2_ , *r* ≥ 0.7) [28]. However, noise included reports of traffic and neighbour noise which were not strongly associated. Interestingly high air pollution levels were not associated with the other two derived factors, “violent crime and physical disorder”, and “crowding and poor access to resources”. Neither was air pollution associated with area socioeconomic position or area educational attainment suggesting that social and environmental stressors are not always consistently geographically patterned in urban areas. In summary, there is a range of correlations between environmental noise and air pollution indices with the highest correlations close to road traffic sources.

### 3.2. Noise Exposure, Air Pollution and Cardiovascular Health Outcomes

#### 3.2.1. Studies Performed on the Adult Population

Most studies in the adult population focus on middle-aged and older samples as these age groups are most at risk of cardiovascular disease. The youngest participants in these studies were 15 years old [17].

#### 3.2.2. Hypertension and Atherosclerosis

Measured hypertension was classified in these studies as a systolic blood pressure greater than 140 mm Hg and a diastolic blood pressure greater than 90 mm Hg. Seven studies of measured raised blood pressure in adults or hypertension were reviewed here [5,6,7,8,9,10,11]; five studies found associations of road traffic noise with raised blood pressure or hypertension adjusting for air pollution either NO_2_ or PM_2.5_ (Table 1). But often these associations were confined to subgroups: restricted to 45–55 year olds [6], older men [8], just for systolic blood pressure not diastolic pressure [11]. One study found associations with night time railway noise [8]. Three studies found associations with air pollution [7,9,10], adjusting for noise exposure. One road traffic noise study found that NO_2_ exposure was associated with SBP in participants exposed to higher traffic noise levels, above the median, (β = 2.28; 95% CI: 0.58, 3.97) and not in those exposed to lower levels (β = −0.79; 95% CI: −2.73, 1.15), *p* for interaction = 0.007. This was also found for L_night_ ≥ 55 dB(A) (β = 1.82; 95% CI: 0.56, 3.07) compared with those exposed to lower noise levels (β = −0.39; 95% CI: −2.17, 1.39), *p* for interaction = 0.03 [10]. Interactions between air pollution and noise exposure may mean that at higher traffic noise levels NO_2_ is more representative of near road pollution, rather than background levels, measured in this study, as daily means at an urban background station [10].

One study assessed thoracic aortic artery calcification as a measure of atherosclerosis, a risk factor for coronary heart disease [12]; both L_night_ and PM_2.5_ exposure were associated with increased subclinical atherosclerosis. In summary, both road traffic noise and air pollution have been associated with raised blood pressure although the results are not always consistent across the population.

#### 3.2.3. Cardiovascular Morbidity

Six studies of myocardial infarction were reviewed [13,14,15,16,17,18,19], of these four also included stroke or cerebrovascular disease [4,16,17,18]. There is also one additional study that only considered stroke [19]. Cardiovascular disease was measured by registry or national records (including fatal myocardial infarction) in 4 studies [4,13,14,15], by hospital admissions in 3 studies [4,17,18], and by self-report in one study [16]. Three studies of road traffic noise found associations with myocardial infarction [13,14,15], although one study of women measured noise only by distance to major roads and the associations were most prominent in those moving closer to major roads [15]. One study did not find effects of road traffic noise after full confounding factor adjustment [17]. All four studies also showed weak associations between air pollution (NO_x_ and NO_2,_ elemental carbon) and myocardial infarction [13,14,15,17]. The stroke study found effects of both road traffic noise and air pollution on stroke but only air pollution, not noise, was related to incidence of fatal stroke [19]. The strongest associations were found for the combination of noise and air pollution although interactions were not statistically significant, for higher noise levels (*p* = 0.67), for higher air pollution levels (NO_2_, *p* = 0.34) [19]. These findings are interesting in the light of acute exposure studies to air pollution (PM_2.5_, Black Carbon) from road traffic where interactions have been shown with noise levels above 65.6 dB(A) showing increased associations with heart rate variability in young healthy adults aged 19 to 32 years [29]. 

Three studies found effects of aircraft noise on self-reported myocardial infarction [16] and CVD hospital admissions [4,18]; these associations were maintained with adjustment for PM_10_ [4], NO_2_ [16] and PM_2.5_ and ozone [18].

#### 3.2.4. All-Cause and Cardiovascular Mortality

Five studies of noise, air pollution and mortality were reviewed [2,3,4,20,21]. One road traffic noise study found an association with black smoke and cerebrovascular and heart failure mortality not reduced by adjustment for noise exposure while the effect of road traffic noise on heart failure death became non-significant after adjusting for black smoke [2]. One study found associations of road traffic noise with mortality that were diminished but still remained significant after adjusting for PM_2.5_ and NO_x_ [21]. Two studies found associations of aircraft noise with mortality that were unaffected by adjustment for PM_10_ [3,4].

#### 3.2.5. Summary of Noise, Air Pollution and Cardiovascular Outcomes

In a recent review of nine publications up to 2012 [30], Tétrault found that the point estimates of the association between traffic noise and cardiovascular disease changed less than 10% after adjustment for air pollution with the exception of three studies [2,5,6]. There were similar findings for two air pollution and cardiovascular disease studies adjusted for noise exposure [2,8]. The correlation between noise and air pollution ranged widely between 0.16 and 0.72 in these studies; the conclusion was that the strength of the correlation did not affect the strength of the confounding of noise studies by air pollution or vice versa. Similarly, it was judged that the quality of the study or the exposure assessment did not influence these confounding effects. In this current review, which is able to include studies published since the Tétrault review, there is good evidence of traffic noise effects on cardiovascular outcomes that are only minimally diminished, on the whole, by air pollution. Similar associations have been found in men and women [2,6,20].

All of these studies are of moderately high quality, have good assessment of noise and air pollution exposure and take into account large numbers of confounding factors. Response rates are not always high and the representativeness of some studies may be questionable but it unlikely that this biases the associations between noise and air pollution and the CVD outcomes except in the fewer longitudinal studies. The focus of most of these studies is on noise but they also demonstrate independent effects of air pollution on these cardiovascular outcomes.

#### 3.2.6. Noise Exposure, Air Pollution and Cognitive Effects and Mental Health

There have been few studies that have simultaneously considered the effects of air pollution and noise on cognition and mental health in adults [31]. In a review of 15 studies of adults greater than 18 years, there was a tendency for air pollution exposure to be associated with cognitive decline and noise exposure to be associated with depression and anxiety disorders. Partly this may be that cognitive decline has been studied less in relation to noise exposure but also air pollution and noise may have differing effects on the nervous system. Many of the studies reviewed in this article had methodological problems. For example, in some studies covariates were not adequately adjusted for and the potential overlap between mood and cognitive outcomes was not considered. There is scope for further research on both cognitive and psychiatric disorders examining noise and air pollution simultaneously. In summary, the results are suggestive of effects of air pollution and environmental noise on cognitive function and mental health but more research needs to be done before any conclusions can be drawn.

### 3.3. Studies Performed on Children and Infants

#### 3.3.1. Noise Exposure, Air Pollution and Reproductive Outcomes

Road traffic noise was associated with term birth weight in 68,238 singleton births adjusting for sex, ethnicity, parity, birth month and year and income and education (mean difference = −19 95% CI −23,−15) [32]. The association with noise remained unchanged after further adjustment for air pollution measured using temporally adjusted land use regression models. Conversely air pollution estimates decreased after adjustment for noise. In a study of access to greenness, noise and air pollution, noise exposure and air pollution did not influence the association of greenness with birth outcomes although proximity to greenness did reduce the effect of noise by 50% [33]. Greenness within 100m of residence is associated with increased risk of babies having higher term birth weight, and less risk of being small for gestational age and term birth weight. Individual and area socioeconomic status (SES) variables attenuated the association of proximity to greenness and the birth outcomes.

#### 3.3.2. Noise Exposure, Air Pollution and Cognitive Outcomes

Two studies have considered the joint effects of noise and pollution on cognitive outcomes in 9–11 year old children using data from the UK and Dutch samples of the Road traffic and Aircraft Noise exposure and Children’s cognition and Health: exposure-effect relationships and combined effects (RANCH) study, respectively [22,23]. Both studies show associations of aircraft noise with cognitive effects including reading comprehension, memory and attention measured with different assessments after taking air pollution into account. One study shows effects of NO_2_ on memory span length [23] while the other [22] shows no effects of air pollution on reading comprehension, memory and attention. Generally there are too few studies to definitively judge on whether air pollution or noise, when assessed in the same study, is more prominently associated with childhood cognition. However, this is against a background of a large amount of studies demonstrating associations of noise with cognition and there may be quite different pathways for noise and air pollution effects on the brain [34].

#### 3.3.3. Noise Exposure, Air Pollution and Blood Pressure

Two studies have compared the associations of environmental noise and air pollution with blood pressure in children [22,24]. One study of road traffic, from Bilenko *et al*. found associations of air pollution with diastolic blood pressure but no associations with noise [24] while the other study from Clark *et al*. of aircraft and road traffic finds no associations of either pollutant with blood pressure [22]. The low level of noise in the Bilenko study may account for the lack of noise associations with blood pressure but it had more detailed measures of air pollution since birth including NO_2_, PM_10_ and PM_2.5_ [24]. The Clark *et al.* study [22] had only moderate levels of NO_2_ and the sample size was relatively small, with blood pressure measurements in only half the original UK sample from the RANCH study which may partly account for the lack of positive associations between the exposures and blood pressure. These two studies are in the context of a larger number of studies examining road traffic noise alone on children’s blood pressure which do show moderate consistency in elevation of systolic blood pressure of between 2–5 mm Hg [35]. For example, the study which demonstrated a 4–5 mm Hg difference in systolic blood pressure, had a mean noise level in the high noise area of 66.9 ± 5.3 dB(A) and the mean noise level in the low noise area of 55.7 ± 2.8 dB(A). The implications for adult cardiovascular health of such elevation in blood pressure are unknown but they are indicative of a physiological response to noise in children.

## 4. Discussion

This paper focuses on noise exposure, air pollution and health. This next section discusses the contribution of noise to the burden of disease relative to other environmental pollutants including air pollution.

### 4.1. Reviews of Environmental Stressors and Health

#### 4.1.1. Noise and Environmental Burden of Disease

The overall burden of disease from a range of environmental pollutants has been assessed and put into context to assess the comparative contribution of different pollutants. Most of these reviews of the impact of environmental stressors include not only air pollution and noise but also a range of chemical contaminants of the external environment and radioactivity.

#### 4.1.2. National Studies

Initial efforts to assess the burden of disease from environmental pollutants in the Netherlands population used the fourth Dutch National Environmental Outlook to integrate estimates of life expectancy, quality of life and number of people affected, to assess the years of healthy life lost due to environmental pollutants [36]. They estimated that particulate air pollution accounted for 60% of the Disability Adjusted life years lost (DALYs) attributable to environmental factors whereas noise accounted for 24% and indoor air pollution 6%. They estimated that the total mortality from particulate air pollution amounted to 169,000 DALYs while 17,700 DALYs were attributed to noise annoyance, 10,990 to sleep disturbance from noise, 50 DALYs to ischaemic heart disease (IHD) from noise and 10 DALYs to mortality related to noise [36]. This study was carried out before the recent blossoming of cardiovascular research on noise.

Years of cardiorespiratory life lost due to environmental noise (road, rail and air traffic) and air pollution were assessed for Switzerland in 7.8 million people using data from 2010 [37]. Environmental noise was measured as L_den_ above a threshold of no effect of 48 dB(A), air pollution was measured as PM_10_. In terms of exposure, 84% of 7.8 million residents were exposed to road traffic noise greater than 48 dB(A). Transportation sources contributed 26% of the total load of PM_10_. In 2010 it was estimated that there were 6000 years of life lost due to noise, largely from cardiovascular disease. At the same time 14,000 years of life lost were estimated for air pollution. The contribution of road traffic noise to years of life lost from cardiovascular disease was assessed as 78%. Morbidity was assessed as hospital days for IHD, stroke and hypertensive disease. 4700 days for cardiovascular disease (CVD) and 4000 days for respiratory disease were assessed as due to air pollution, and 13,800 days for IHD, 4600 for stroke and 4100 days for hypertensive disease were due to noise.

#### 4.1.3. European Studies

In 2011 WHO Europe published the burden of disease from environmental noise [38]. Based on noise exposure assessment, the distribution of exposure and existing exposure response relationships, DALYs lost from environmental noise were calculated for EU member states and other Western European countries. 61,000 DALYs were attributed to ischaemic heart disease based on hypertension and IHD outcomes, 45,000 DALYs to cognitive impairment in children and young people, aged 7–19 years, 903,000 DALYs to sleep disturbance for people living in towns with more than 50,000 inhabitants, 22,000 DALYs to tinnitus, and 654,000 DALYs for annoyance.

The environmental burden of disease in European countries project assessed burden of disease from nine environmental risk factors in six European countries [39]. Road, rail and air traffic noise were included and linked to health endpoints of severe sleep disturbance and ischaemic heart disease. DALYs were presented as population-weighted, non-discounted and non-age weighted annual averages per million people. Estimates were derived from European Noise Directive (END) reporting from 2007, of agglomerations with >6 million vehicles per year, railways with >60,000 trains per year and airports >50,000 flights per year- which is likely to be an underestimate of the magnitude of effects. Only exposure levels 50 dB(A) L_night_, 50 dB(A) L_den_ were available so lower noise levels could not be assessed [39]. The relative population-weighted contribution of traffic noise was 8% compared to 68% for particulates; traffic noise accounted 400–1500 DALYs per million people. This was substantial because of high population exposure despite relatively small disability weights for severe sleep disturbance (0.07). The DALYs attributed to noise were more than those attributed to lead (100–900), ozone (30–140) dioxins (200–600) DALYs.

#### 4.1.4. Willingness to Pay Studies

Another way of assessing the burden of environmental stressors, increasingly of interest to governments, is willingness to pay (WTP) for pollutant exposure. In a study of 5243 people exposed to air pollution and 5251 noise exposed people, gender, education, and financial position did not affect willingness to pay [40]. Increased environmental concerns, noise concerns, noise sensitivity and ability to relax in noisy situations did affect willingness to pay but not awareness of current health risks of noise exposure. The mean estimates to avoid road-traffic noise effects for the three vignettes were: €90 pp/y for general health risks, €100 pp/y for a 13% increase in severe annoyance, and €320 pp/y for a combined-risk scenario related to an increase of a noise level from 50 dB(A) to 65 dB(A). Generally people were willing to pay more for better air quality than noise [40]. This reflects the individual variability in tolerance of exposure to noise that cannot easily be taken into account in burden of disease calculations.

### 4.2. Summary of Findings, Mechanisms and Potential Interventions

In general the studies reviewed suggest independent associations of environmental noise, from road traffic, aircraft and rail, and air pollution with cardiovascular outcomes and mortality and evidence for noise impacts on cognitive outcomes in children and for air pollution too. In terms of burden of disease European studies demonstrate that air pollution leads the environmental factors especially in relation to mortality [39]. Nevertheless, environmental noise comes second in terms of burden of disease and arguably is responsible for more disturbance of quality of life. Environmental noise is also responsible for more life years lost than other significant environmental pollutants such as lead, ozone and dioxins [39].

In terms of designing health interventions in relation to transportation why should planners consider noise in addition to air pollution? First, the distribution of noise and air pollution may be different. The correlations between noise and air pollution vary enormously between studies but are generally found to be moderate [30]. Although these may be influenced by factors related to pollutant measurement they also reflect the differing dispersion patterns of the two pollutants. Noise is influenced by intervening barriers and buildings, air pollution by meteorological conditions [27]. Thus different people may be affected by the same transport source. Secondly, the evidence on the mechanisms for the two health effects differs between the two pollutants. For instance, black carbon exposure is thought to lead to oxidative stress and inflammation but also to direct effects on the cardiovascular system leading to myocardial ischaemia [2]. Particles may activate the sympathetic nervous system through stimulation of the pulmonary reflex [30]. Noise exposure is thought to activate stress mechanisms with stimulation of secretion of “stress hormones” such as cortisol and catecholamines. Noise may also cause short term vasoconstriction and in the longer term atherosclerosis due to metabolic changes [11]. There may be some overlap in mechanisms as oxidative damage has also been observed after traffic noise exposure in mononuclear blood cells in laboratory conditions [41]. For effects on the brain it is postulated that particles may activate pro-inflammatory cytokines in human macrophages initiating an inflammatory response and oxidative stress and fine particles may be directly absorbed into the nervous system through the olfactory bulb [31]. In contrast noise effects on mood disorders may result from activation of physiological arousal and stress pathways. In summary, the evidence suggests that noise and air pollution may be affecting different aspects of cardiorespiratory health. Moreover, while air pollution may affect the lungs, noise may lead to annoyance and sleep disturbance—thus people are affected in quite disparate ways by noise and air pollution.

Interventions that limit both air pollution and noise would be most beneficial but not all interventions are equally effective for both exposures and some interventions for road traffic may reduce one exposure at the expense of another [42]. Curran et al suggest that two strategies of increasing separation between vehicles and the residential population and reducing the overall volume of vehicles are the most effective strategies for reducing both pollutants but changes in fuel, vehicle speed or driver behavior may have more diverse effects reducing one pollutant while increasing the other because the sources of each pollutant within the vehicle differ [43]. Interventions that tackle the exposures at source are generally more effective than interventions that modify the conditions of the receiver, such as sound insulation. Land use planning, incorporating strategies to reduce overall individual vehicle use may ultimately be most effective in reducing both noise and air pollution [43].

### 4.3. Limitations of the Studies and the Review

The review was not intended to be comprehensive and it is possible that some important studies have been left out although this is unlikely to change the overall summary findings of the paper. The quality of most recent studies in this field is high with careful measurement and modelling of noise and air pollution, large sample sizes, an increasing focus on “objective” health outcomes and extensive adjustment for confounding factors. There are some methodological weaknesses that limit the conclusions than can be drawn on the relative importance of noise and air pollution.

Exposure misclassification is an important source of error in air pollution [42] and noise modelling. People moving during the study exposure period leading to changes in exposure are often not accounted for, duration of exposure may not be measured accurately [2], and exposure to several sources of noise [13] may not be fully accounted for, especially additional occupational exposure. Many studies assume people stay in their homes all day which may lead to exposure misclassification. Study selected air pollution indicators are not always specific indicators of road traffic emissions [42] and may differ in toxicity [44]. Temporal misalignment of exposure measurement and health outcomes may lead to an underestimation of the magnitude of effects [45]. A lack of variation in air pollution exposure may explain the lack of effect of air pollution on hypertension [6]. Outdoor residential noise exposures which are usually modelled to represent individual noise exposure are not always associated with personal exposures for noise that may reflect indoor exposure. Some studies, for want of anything better still use to distance to roads as a proxy measure of noise which is a crude indicator [14]. Despite the size of many studies some still lack sufficient power to test for interactions between noise and air pollution [13].

Adjustment for confounding is a pertinent issue in environmental studies where the huge variety of different influences on exposure and health may make effects of noise and air pollution difficult to detect. Confounding may depend on exposure assessment, categorisation of exposure thresholds, study design, the choice of health outcome, and other urban characteristics [42]. The varied results of air pollution and noise on hypertension may result from residual confounding. There may also be negative confounding by noise representing people taking precautions to reduce noise levels such as closing windows. Factors such as bedroom location in relation to noise exposure, closing windows, sound insulation measures, presence of hearing impairment, may also be moderating rather than confounding factors that tend to be included in only the most recent studies. Simultaneous adjustment for traffic intensity in road traffic noise studies may be over-adjustment partly accounting for the effects of traffic noise [2]. There are also limitations in burden of disease studies which rely on exposure-response relationships which have a degree of uncertainty and may not be generalizable across large populations. Additionally, the availability and quality of health data that contribute to burden of disease studies varies, and disease definitions are not constant across component studies. All of these limitations contribute to the variations in the magnitude of estimated effects of environmental stressors. Nevertheless, in broad terms they do not affect the ranking of the importance of stressors.

### 4.4. Further Research

There is scope for further research. Birth cohorts offer many opportunities for taking into account life course factors but modelling exposures across larger areas can be challenging. Objective noise measurements in accordance with the European Noise Directive (END) using noise propagation modelling are needed with information on non-residential exposure, time activity patterns, insulation of buildings, widow opening behaviour, and position of bedrooms in relation to noise source [44]. More standardisation of indicators of air pollution is needed; black carbon, NO_x_ and ultrafine particles might be more relevant than currently used indicators [27].

## 5. Conclusions

There is good evidence from large population studies that environmental noise from road traffic and aircraft is associated with cardiovascular morbidity and mortality independent of the association with air pollution. There may be both independent mechanisms and common mechanisms involving methylation for these associations of environmental exposures with health. Environmental planning and policy should take both exposures into account when assessing environmental impacts.
